# Remimazolam for preventing sevoflurane-induced emergence delirium after pediatric laparoscopic inguinal hernia repair: a placebo-controlled randomized clinical trial

**DOI:** 10.1016/j.jped.2025.101423

**Published:** 2025-08-22

**Authors:** Yiwen Lu, Guangbin Liang, Yan Lu, Jingjing Wang, Jianhang Mo, Zhiyuan Chen, Ruizhao Shao, Kui Hu, Ping Pang, Xiaoxia Gu

**Affiliations:** a Affiliated Hospital of Guangdong Medical University, Anesthesia Operation Center, Zhanjiang City, Guangdong Province, China; b Maoming People's Hospital, Department of Anesthesiology, Maoming City, Guangdong Province, China

**Keywords:** Remimazolam, General anesthesia, Laparoscopic surgery, Emergence delirium, Pediatric inguinal hernia

## Abstract

**Objective:**

To investigate the effectiveness and safety of remimazolam in preventing the emergence of delirium in children undergoing pediatric laparoscopic inguinal hernia surgery under combined intravenous and inhalation anesthesia.

**Methods:**

A total of 184 pediatric patients aged 3–14 years who were undergoing laparoscopic inguinal hernia surgery were included. Patients were randomly assigned to receive either 0.1 mg/kg remimazolam (experimental group, *n* = 92) or 0.9 % normal saline (control group, *n* = 92) after the procedure. The primary outcome was the incidence of emergence delirium, which would manifest as a difference in the pediatric anesthesia emergence delirium (PAED) score between the two groups. The secondary outcomes included postoperative pain severity, duration of postanesthesia care unit (PACU) stay, sufentanil usage, parental satisfaction, mean arterial pressure (MAP), blood oxygen saturation (SpO₂), and heart rate (HR).

**Results:**

The experimental group showed significantly lower overall PAED scores (mean difference = −2.28, 95 % CI:2.73 to −1.82; Cohen's *d* = 1.12, *p* < 0.001) and emergence delirium incidence (7.61 % vs 41.30 %; risk difference = 33.69 %, 95 % CI 21.60 % to 45.10 %; relative risk = 0.18, 95 % CI 0.09–0.37; *p* < 0.001). They also showed better hemodynamic stability (lower MAP/HR, higher SpO₂), reduced pain, shorter PACU stay, less sufentanil use, and higher parental satisfaction (all *p* < 0.05).

**Conclusion:**

Administration of 0.1 mg/kg remimazolam after pediatric laparoscopic inguinal hernia surgery could contribute to preventing the development of emergence delirium by smoothing hemodynamic changes and alleviating postoperative pain.

## Introduction

Inguinal hernias are one of the most common congenital anomalies seen by pediatric surgeons. The overall incidence ranges from 0.8 % to 5.0 % in full-term infants and up to 30.0 % in low birth weight and premature infants [1]. Without timely treatment, inguinal hernia incarceration or intestinal perforation may occur, and in severe cases, permanent inguinal hernias in children could affect reproductive function. However, pediatric inguinal hernias are usually not self-healing, and surgery is the most effective treatment [2]. Laparoscopic surgery for pediatric inguinal hernias has the advantages of shorter operation times, smaller incisions, and shorter hospital stays [3]. However, laparoscopic surgery may be associated with a greater risk of developing emergence agitation (EA) [4], which could lead to complications such as wound dehiscence, bleeding, or the need for reoperation, thereby compromising surgical success [5].

EA is a general term that includes emergence delirium (ED), pain and several other factors [6]. ED is an acute confusion state that occurs during recovery from anesthesia; patients with ED may present with disorientation, hallucination, restlessness, and purposeless hyperactive physical behavior [7,8]. EA and ED in the PACU are strong predictors of postoperative delirium, which is associated with prolonged hospital stays, increased morbidity (e.g., pulmonary complications), increased mortality, and the need for institutionalization of patients as adults [8]. Therefore, preventing the development of EA is of great importance for the postoperative management of children with inguinal hernias.

Remimazolam, a novel benzodiazepine sedative-hypnotic drug, has the advantages of greater sedative efficacy, greater controllability, a shorter duration of action, and fewer side effects than other sedatives [9,10]. Additionally, it has a minimal effect on blood pressure fluctuations and respiratory movements. Moreover, it does not cause liver or kidney damage because it is rapidly hydrolyzed into inactive metabolites [11]. Therefore, remimazolam has been widely adopted in routine practice. Remimazolam has been reported to reduce the excitability of the central nervous system by enhancing the function of GABA receptors [12,13]. In addition, in children who underwent tonsillectomy and adenoidectomy, intravenous remimazolam at the end of surgery was effective in alleviating sevoflurane-induced ED [14]. While remimazolam has demonstrated efficacy in reducing ED following tonsillectomy procedures, critical knowledge gaps remain regarding its application in laparoscopic inguinal hernia repair. Therefore, this study aimed to evaluate the effectiveness and safety of remimazolam in preventing the emergence of delirium in children undergoing pediatric laparoscopic hernia surgery under combined intravenous and inhalation anesthesia. The authors hypothesize that remimazolam may reduce the incidence of EA after laparoscopic inguinal hernia surgery.

This study specifically addresses two unmet needs in pediatric anesthesia: (1) the lack of data on remimazolam's effects during pneumoperitoneum-induced stress responses, which may exacerbate EA risk; and (2) limited safety profiles when combined with opioid-sparing techniques. By investigating a standardized 0.2 mg/kg remimazolam dose administered at induction, the authors aim to establish evidence-based guidelines for EA prevention in this understudied surgical context while evaluating recovery quality and hemodynamic stability.

## Methods

### Study design and participants

This parallel, randomized, single-center trial was performed at the Hospital. The Ethics Committee of the Hospital approved this study under reference number PJ2021–126. Written informed consent was obtained for the participation of all patients before their inclusion in the study. Moreover, the design and implementation of this study were guided by the ethical principles of the Declaration of Helsinki and good clinical practice principles [15]. The study was registered with the Chinese clinical trial registry (ChiCTR2000038840 at *http://www.chictr.org.cn*; date, 5 October 2020).

Patients who were diagnosed with unilateral or bilateral inguinal hernias by imaging examinations and satisfied the indications for laparoscopic surgery were included in this study. The exclusion criteria for inguinal hernia patients were as follows: 1) contraindications to anesthesia or surgery; 2) coagulation dysfunction or autoimmune diseases; 3) congenital diseases, such as congenital heart diseases or pulmonary dysfunction; or 4) the need for emergency surgery. Moreover, the withdrawal or termination criteria for participants were as follows: 1) the participants (family members) decided to withdraw midway; 2) serious adverse events occurred in the participants; or 3) the investigators violated the research protocol.

### Randomization and masking

Randomization was performed via a random-block method. All participants were assigned a number, starting from 001. The same number was not reused. Every 4 patients with adjacent numbers were subsequently grouped into a block in numerical order. That is, patients with numbers 001–004 were in one block, whereas those with numbers 005–008 were in another block. Patients in blocks were randomly divided into an experimental group (Group A) and a control group (Group B). Therefore, there were 6 different arrangements for the assignment of patients in one block: AABB, ABAB, ABBA, BBAA, BABA, and BAAB. Six consecutive numbers were selected from the random number table (the same number was not selected twice), which could correspond to the 6 arrangements in numerical order. For example, if the selected consecutive numbers were 6, 3, 2, 7, 1, and 9, the corresponding arrangements would be the 4th, 3rd, 2nd, 5th, 1st, and 6th permutations.

### Study procedures

All patients fasted for 8 h. All patients received no premedication and were accompanied by a parent to the operating room, where an anesthetist, blinded to the group allocation, induced general anesthesia. Standard monitoring, including peripheral oxygen saturation, electrocardiography, and noninvasive blood pressure, was conducted on all subjects. Propofol (1.5–2 kg/mL) was injected intravenously as the sedative premedication. The children were subsequently sent to the operating room. Combined intravenous and inhalation anesthesia was performed. Specifically, 6–7 % sevoflurane in oxygen at 6–7 L/min was used to induce inhalational anesthesia via a facemask. Moreover, 0.2 μg/kg fentanyl and 0.1 mg/kg atracurium cisatracurium were administered intravenously. When a sufficient depth of anesthesia was achieved (that is, the inhalation concentration of sevoflurane reached 2.7 MAC), tracheal intubation was performed. Anesthesia was maintained by 2 %−3 % sevoflurane and 0.15–0.2 µg/kg/min remifentanil. Moreover, the end-tidal carbon dioxide partial pressure (PetCO_2_) was maintained at 35–45 mmHg, which was achieved by adjusting the tidal volume and respiratory rate. Immediately following surgical closure, both sevoflurane and remifentanil were discontinued. At this timepoint (prior to extubation but after anesthesia cessation), the children were randomly administered 0.2 mg/kg remimazolam[14] diluted in 10 mL saline (the experimental group, *n* = 92) or the same volume of saline (the control group, *n* = 92). All the children were given 0.1 mg/kg dexamethasone to prevent nausea and vomiting. When patients were hemodynamically stable, they were sent to the postanesthesia care unit (PACU). In addition, 0.1 μg/kg sufentanil was given for postoperative analgesia. If delirium occurred, propofol 1 mg/kg was administered as a rescue medication and repeated if the delirium did not subside. Removal of the tracheal tube (commonly known as "extubation") was typically performed in the PACU. All syringes were labeled only with patient ID numbers by an independent anesthesiologist not involved in outcome assessment.

Demographic data and clinical characteristics such as age, height, weight, and ASA class were recorded before the surgery. Two research assistants recorded the data needed.

### Outcome measures

The primary endpoint of the study was the incidence of agitation during the recovery period at 0 min, 10 min, 20 min and 30 min after extubation, which could be represented by the score obtained from the Pediatric Anesthesia Emergence Delirium (PAED) scale[6] This scale includes 5 items with a total score of 20. Additionally, the score positively correlates with the severity of agitation (Supplementary Table 2). Emergence delirium was defined as a global PAED score ≥ 10. The secondary outcomes were indicators related to the surgery, which included the length of the surgery, extubation time, length of stay in the PACU, mean arterial pressure (MAP), blood oxygen saturation (SpO_2_), heart rate (HR), pain severity, and parental satisfaction. The pain severity and the MAP, SpO_2_, and HR of all patients were measured every 10 min during the first 30 min after extubation. All outcome assessors remained successfully blinded throughout data collection.

The degree of pain severity was assessed using the face, legs, activity, cry, and consolability (FLACC) scale [16]. The total score of this scale is 10, and the score is positively associated with the severity of pain. Children who were awake were observed for at least 1–2 min to determine the level of pain severity, and children under anesthesia were observed for >2 min. The PAED and FLACC scales were evaluated by trained caregivers or anesthesiologists.

Parental satisfaction was evaluated using the 5-point Likert scale, whose output was very satisfied, satisfied, general, dissatisfied or very dissatisfied. Parental satisfaction was assessed 1 day before discharge, and the parental satisfaction rate was determined as the proportion of parents who selected "very satisfied" or "satisfied" out of the total number of parents. Satisfaction rate (number of very satisfied+number of satisfied)/total number of parents × 100 %.

### Statistical analysis

The study was adequately powered to display a difference that could reach statistical significance in the incidence of emergence agitation between the experimental group and the control group. The incidence of pediatric anesthesia emergence delirium (PAED) caused by sevoflurane anesthesia is approximately 50 % [17]. The authors hypothesize that a 35 % reduction in the incidence of EA between the two groups would be clinically significant (a threshold selected for its clinical relevance in: (1) reducing rescue interventions;[14] (2) aligning with effect sizes of established alternatives [18]). Therefore, 48 patients per group were required to ensure a reliable conclusion at a 5 % significance level and 80 % power, with a superiority margin of 4 % according to a two-sided test. Sample size estimation was performed with PASS 11.0 software.

Normality was tested via the Shapiro‒Wilk test. Normally distributed data are presented as the means ± standard deviations and were compared with independent sample *t* tests. Nonnormally distributed data are reported as medians (25th percentile, 75th percentile) and were examined via the Mann‒Whitney U test. The chi-square test was applied for categorical variables, whereas the Wilcoxon rank-sum test was applied for rank data. Two-way ANOVA was used for the analysis of continuous hemodynamic data. The PAED/FLACC scores were analyzed using a two-way repeated measures ANOVA, with post hoc tests performed using the Sidak method. *P* < 0.05 was considered to indicate a significant difference. Differences in baseline characteristics between groups were assessed using the Standardized Mean Difference (SMD). For continuous variables, SMD was calculated as: $\;\text{SMD}=\frac{|\text{Media}n_{1}-\text{Media}n_{2}\;|}{\sqrt{(\text{IQR}1^{2}+\text{IQR}2^{2})/2}}$, where Interquartile Range (IQR) =75th percentile-25th percentile. For categorical variables, SMD was computed as:$\;\text{SMD}=\frac{|p_{1}-p_{2}\;|}{\sqrt{p_{\text{pooled}}\times \left(1-p_{\text{pooled}}\right)}}$, where p_pooled=_(n1p1+n2p2)/(n1+n2), with p1 and p2 being the proportions of a specific category in each group.An SMD ≥ 0.2 was considered indicative of meaningful imbalance, warranting discussion of its potential clinical significance.All the statistical analyses were performed with SPSS 25.0 software.

## Results

According to the inclusion and exclusion criteria, 184 participants were recruited for this study. The differences in sex, age, weight, height, and ASA grade between the experimental group (treated with remimazolam) and the control group (treated with saline) were not significant (SMD < 0.2; Table 1). In addition, there was no significant difference in extubation time between the two groups (*p* > 0.05; Table 2). In particular, patients in the remimazolam group demonstrated significantly improved recovery outcomes, including a 12.32-minute reduction in PACU stay time (95 % CI: 10.67–13.97 min; *p* < 0.001) and 0.57 μg lower sufentanil consumption (95 % CI: 0.19–0.94 μg; *p* = 0.003) compared to controls (*p* < 0.05; Table 2).

**Table 1:** Comparison of baseline data between the two groups.

	Control group (*N* = 92)	Experimental group (*N* = 92)	SMD-value
Sex, n ( %)			
Female	21 (22.83 %)	15 (16.30 %)	0.165
Male	71 (77.17 %)	77 (83.70 %)	
Age (years)	5 (4,6)	5 (4,7)	0.000
Height (cm)	118.5 (107.3127.8)	115.5 (104.0124.8)	0.145
Weight (Kg)	16.50 (14.00,21.00)	17.00 (14.73,19.90)	0.081
ASA Classification, n ( %)			
ASA I	68 (73.91 %)	73 (79.35 %)	0.129
ASA II	24 (26.09 %)	19 (20.65 %)	
Distribution, n ( %)			
Unilateral	88 (95.65 %)	68 (93.48 %)	0.096
Bilateral	4 (4.35 %)	24 (6.52 %)

**Table 2:** Analysis of surgery-related indicators between the two groups.

	Control group (*N* = 92)	Experimental group (*N* = 92)	p-value
Operation time (min)	32.15 ± 8.39	27.97 ± 6.14	<0.001
Extubation time after operation (h)	17.75 ± 9.18	18.98 ± 9.87	0.381
Postanesthesia care unit stagnation time (min)	39.83 ± 6.19	27.51 ± 5.10	<0.001
The sufentanil usage (μg)	9.00 (7.00,11.00)	8.00 (7.00,10.00)	0.003

**Figure 1:**
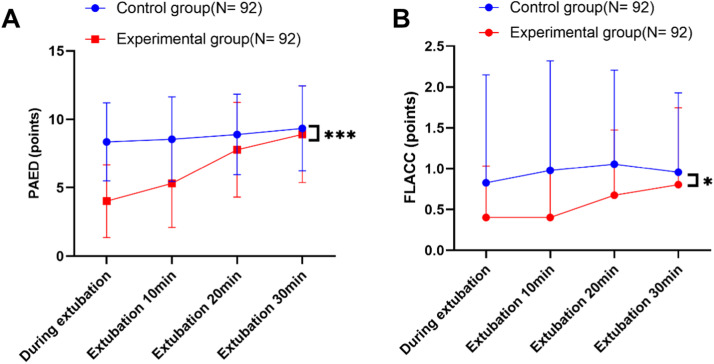
Analysis of emergence agitation and pain intensity during extubation between the two groups. *Note:* A: PAED scores during extubation; B: FLACC scores during extubation. Data were expressed as mean ± standard deviation, Two-way repeated measures ANOVA was used for comparison between groups, and the Sidak method was used for post hoc tests. * *p* < 0.05, *** *p* < 0.001.

Two-way repeated measures ANOVA demonstrated significantly lower PAED scores in the remimazolam group compared to controls (mean difference = −2.28, 95 % CI: −2.73 to −1.82; Cohen's *d* = 1.12, *p* < 0.001) (Figure 1A). Post hoc Sidak tests confirmed significantly lower scores in the experimental group at 0 min (mean difference: −4.33, 95 %CI: −5.13 to −3.53, *p* < 0.001), 10 min (−3.23, −4.15 to −2.31, *p* < 0.001), and 20 min (−1.11, −2.05 to −0.18, *p* = 0.020), but not at 30 min (*p* = 0.383) (Table 3). Emergence delirium occurred in 7 of 92 (7.61 %) patients receiving remimazolam versus 38 of 92 (41.30 %) patients receiving saline (risk difference 33.69 % [95 % CI, 21.60 % to 45.10 %], relative risk = 0.18 [95 % CI, 0.09 to 0.37]; *p* < 0.001).

**Table 3:** Analysis of each detection index in the two groups.

Index	Control group (*N* = 92)	Experimental group (*N* = 92)	p-value
**PAED** (points)			
During extubation	8.35 ± 2.86	4.02 ± 2.66	<0.001
10 min	8.55 ± 3.10	5.32 ± 3.23	<0.001
20 min	8.89 ± 2.95	7.78 ± 3.46	0.020
30 min	9.34 ± 3.11	8.91 ± 3.54	0.383
**FLACC** (points)			
During extubation	0(0,1)	0(0,1)	0.041
10 min	1(0,2)	0(0,1)	0.001
20 min	1(0,2)	0(0,1)	0.030
30 min	1(0,2)	1(0,1)	0.257
**MAP** (mmHg)			
Before induction of anesthesia	83.28 ± 5.19	83.19 ± 5.20	0.906
When tube drawing	113.90 ± 6.19	105.29 ± 5.07	0.001
10 min	106.29 ± 6.04	99.21 ± 5.18	0.001
20 min	98.20 ± 5.37	91.03 ± 5.10	0.002
30 min	94.09 ± 4.18	86.90 ± 4.13	0.001
**SpO2** ( %)			
Before induction of anesthesia	99.04 ± 1.08	99.15 ± 0.97	0.468
When tube drawing	96.36 ± 1.35	97.35 ± 1.20	<0.001
10 min	96.39 ± 1.24	97.69 ± 1.38	<0.001
20 min	96.20 ± 1.35	97.40 ± 1.30	<0.001
30 min	97.20 ± 1.45	98.11 ± 1.40	<0.001
**HR (bpm)**			
Before induction of anesthesia	100.40 ± 7.47	101.05 ± 7.20	0.549
When tube drawing	130.7 ± 7.07	125.6 ± 7.29	<0.001
10 min	126.1 ± 6.90	120.5 ± 7.17	<0.001
20 min	120.3 ± 8.57	117.5 ± 8.11	0.024
30 min	115.8 ± 8.87	112.30 ± 8.11	0.006

FLACC scores were significantly lower in the remimazolam group (mean difference = 0.38, 95 %CI:0.24–0.53; Cohen's *d* = 0.82, *p* = 0.026) (Figure 1B), with significant reductions at 0 min (*p* = 0.041), 10 min (*p* = 0.001), and 20 min (*p* = 0.030), but not at 30 min (*p* = 0.257) (Table 3).

As shown in Table 3, there was no significant difference in the MAP, SpO2 or HR between the two groups before anesthesia (*p* > 0.05). Moreover, the MAP and HR of patients in the experimental group at 0 min, 10 min, 20 min, and 30 min after extubation were significantly lower than those of patients in the control group (*p* < 0.05). The SpO_2_ levels of patients in the experimental group at 0 min, 10 min, 20 min, and 30 min after extubation were significantly greater than those of patients in the control group (*p* < 0.05).

With respect to parental satisfaction, the difference in the total satisfaction score between the two groups was not significant (*p* > 0.05). However, there was a significant difference in the proportion of very satisfied parents between the two groups (63.0 % in the experimental group and 37.0 % in the control group). Postoperative nausea or vomiting occurred in 3 of 92 (3.26 %) subjects in the saline group and 2 of 92 (2.17 %) subjects in the remimazolam group (*p* > 0.05). No episodes of bradycardia, hypotension, laryngospasm, or hypoxemia were identified during the study period. The details are displayed in Supplementary Table 1.

## Discussion

The present results demonstrated that the application of 0.1 mg/kg remimazolam after surgery significantly reduced the incidence of agitation during recovery from combined intravenous-inhalation anesthesia. In addition, the postoperative administration of remimazolam significantly increased parental satisfaction and significantly reduced the length of PACU stay without a significant increase in adverse events. Additionally, the authors validated the safety and efficacy of remimazolam.

EA, as a long-term focus, is a common adverse event after general anesthesia. Emergence agitation could cause sympathetic excitation, which could lead to an increase in blood pressure and brain hypoxia [19]. In addition, it can manifest as a psychiatric disorder with involuntary behavior and mania in severe cases [20]. As a result, emergence agitation is detrimental to patient recovery after surgery and may contribute to the development of several postoperative complications. Although the mechanism of emergence agitation is unclear, postoperative pain, certain anesthetics, type and duration of surgery, and preschool age are associated with the occurrence of EA [20,21]. In this randomized clinical trial, approximately half of the participants experienced EA under general anesthesia, which was consistent with the findings of previous studies [22]. The present results demonstrated that remimazolam 0.2 mg/kg administered at surgery completion significantly reduced the incidence of emergence delirium incidence from 41.3 % to 7.6 % in pediatric laparoscopic hernia repair. This effect aligns with previous reports of remimazolam's efficacy in tonsillectomy/adenoidectomy (44 % to 12 %)[14] and meta-analysis data for dexmedetomidine (37 % relative risk reduction) [23]. These consistent outcomes establish remimazolam as an effective alternative for pediatric emergence delirium prophylaxis.

Postoperative pain has been identified as an independent risk factor for the emergence delirium in clinical studies [24], underscoring the importance of pain assessment in delirium evaluation. The FLACC scale - a validated behavioral pain assessment tool -was adopted to assess the pain severity of all pediatric patients. In this study, the authors found that the FLACC scores at 10 min and 20 min after extubation were significantly lower in the experimental group than in the control group, indicating that remimazolam could effectively prevent emergence agitation by reducing the severity of postoperative pain in children who underwent laparoscopic inguinal hernia surgery.

Remimazolam reduces fluctuations in heart rate and blood pressure by inhibiting the activity of the sympathetic nervous system [25]. This pharmacological property, combined with its established safety profile, has led to its widespread adoption in endoscopic procedures. These findings corroborate these advantages, showing significantly lower MAP and HR, along with higher SpO_2_ levels during the initial 30-minute post-extubation period in the remimazolam group. These results collectively support remimazolam's favorable safety profile as an anesthetic agent with minimal cardiovascular stimulation. Additionally, although total parental satisfaction scores did not differ significantly between groups, the higher proportion of "very satisfied" parents in the remimazolam group (63.0 % vs. 37.0 %) likely reflects the marked reduction in emergence agitation, which has a disproportionate emotional impact despite not affecting overall care ratings. The similar incidence of postoperative nausea/vomiting (3.26 % vs. 2.17 %) and absence of cardiorespiratory complications (bradycardia, hypotension, laryngospasm, or hypoxemia) further support remimazolam's safety profile. These findings suggest that while global satisfaction metrics may mask specific benefits, preventing distressing emergence behaviors significantly enhances parental experience without compromising safety.

However, the present study has several limitations. First, the authors did not investigate the correlation between the dose of remimazolam and the development of emergence agitation. Second, this study was performed at a single center. Therefore, the external effectiveness of these results remains unclear. Third, there was no follow-up in this study. Fourth, according to the experimental design, statistical data were not collected at 40 min after extubation. Fifth, the FLACC scale was used alone and not in conjunction with other pain assessment tools, such as the Wong-Baker Facial Expression Pain Rating Scale or the Numerical Rating Scale (NRS). Thus, the long-term effectiveness of remimazolam for EA remains unclear. A multicenter study should be conducted to address these issues. In addition, longer procedures may theoretically increase delirium risk through mechanisms such as prolonged anesthetic exposure or surgical stress. However, in the study design, the randomization process should have equally distributed operative time variations between groups. Future studies could stratify by procedure duration to clarify this relationship.

According to the results of this study, the use of remimazolam at the end of laparoscopic inguinal hernia surgery in children may help reduce postoperative pain and restlessness during postoperative recovery, stabilize patients' vital signs, such as blood pressure and heart rate, and reduce hemodynamic fluctuations caused by surgical stress or pain. This study offers key advantages for clinical practice: (1) Efficiency: rapid recovery (extubation < 10 min) without delaying PACU discharge, (2) Safety: minimal hemodynamic/respiratory effects (MAP/HR fluctuations < 10 %, SpO₂ > 94 %), and (3) Cost-Effectiveness: lower per-dose cost (∼30 %) compared to dexmedetomidine. These benefits, combined with simple bolus administration (no infusion pumps required), position remimazolam as a practical first-line option for pediatric day-case surgery, particularly in resource-limited settings.

## Funding

This study was funded by the Guangdong Provincial Health Commission (Project No A2023280), the Clinical Research Special Fund of Guangdong Medical Association (No. 2024HY-A4019) and the Clinical Research Project of the Affiliated Hospital of Guangdong Medical University (No. LCYJ2020B006).

## Data availability

The data that support the findings of this study are available from the corresponding author upon reasonable request.

## Ethics approval and consent to participate

This study was approved by the Ethics Committee of Affiliated Hospital of Guangdong Medical University (PJ2021–126). All participants were informed and have written the informed consent.

## Retrospectively registered

It was also registered with the Chinese Clinical Trial Registry (ChiCTR2000038840).

## Author's contributions

Yiwen Lu designed the study. Guangbin Liang, Yan Lu, Jingjing Wang, Jianhang Mo, Zhiyuan Chen, Ruizhao Shao and Kui Hu collected the data. Guangbin Liang analyzed the data. Ping Pang interpreted the data. Yiwen Lu, Guangbin Liang, Ping Pang and Xiaoxia Gu wrote and revised the manuscript. All authors have read and approved the final manuscript.

## Conflicts of interest

The authors declare no conflicts of interest.

## Appendix Supplementary materials

Image, application 1

## Footnotes

Supplementary material associated with this article can be found in the online version at doi:10.1016/j.jped.2025.101423.

## References

[1] Abdulhai S., Glenn I.C., Ponsky T.A. (2017). Inguinal Hernia. Clin Perinatol.

[2] Song Y.H., Huang W.J., Xie Y.Y., Hada G., Zhang S., Lu A.Q. (2020). Application of double circular suturing technique (DCST) in repair of giant incision hernias. Ann Transl Med.

[3] Shiraishi T., Tominaga T., Nonaka T., Hamada K., Araki M., Sumida Y. (2021). A learning curve in using organ retractor for single-incision laparoscopic right colectomy. Sci Rep.

[4] Makarem J., Larijani A.H., Eslami B., Jafarzadeh A., Karvandian K., Mireskandari S.M. (2020). Risk factors of inadequate emergence following general anesthesia with an emphasis on patients with substance dependence history. Korean J Anesthesiol.

[5] Kim J.H. (2011). Mechanism of emergence agitation induced by sevoflurane anesthesia. Korean J Anesthesiol.

[6] Sikich N., Lerman J. (2004). Development and psychometric evaluation of the pediatric anesthesia emergence delirium scale. Anesthesiology.

[7] Malarbi S., Stargatt R., Howard K., Davidson A. (2011). Characterizing the behavior of children emerging with delirium from general anesthesia. Paediatr Anaesth.

[8] Lee S.J., Sung T.Y. (2020). Emergence agitation: current knowledge and unresolved questions. Korean J Anesthesiol.

[9] Zhou Y., Hu P., Huang Y., Nuoer S., Song K., Wang H. (2018). Population pharmacokinetic/pharmacodynamic model-guided dosing optimization of a novel sedative HR7056 in Chinese healthy subjects. Front Pharmacol.

[10] Wesolowski A.M., Zaccagnino M.P., Malapero R.J., Kaye A.D., Urman R.D. (2016). Remimazolam: pharmacologic considerations and clinical role in anesthesiology. Pharmacotherapy.

[11] Hu Q., Liu X., Wen C., Li D., Lei X. (2022). Remimazolam: an updated review of a new sedative and anaesthetic. Drug Des Devel Ther..

[12] Chang Y., Huang Y.T., Chi K.Y., Huang Y.T. (2023). Remimazolam versus propofol for procedural sedation: a meta-analysis of randomized controlled trials. PeerJ.

[13] Rogers W.K., McDowell T.S. (2010). Remimazolam, a short-acting GABA(A) receptor agonist for intravenous sedation and/or anesthesia in day-case surgical and non-surgical procedures. IDrugs.

[14] Yang X., Lin C., Chen S., Huang Y., Cheng Q., Yao Y. (2022). Remimazolam for the prevention of emergence delirium in children following tonsillectomy and adenoidectomy under Sevoflurane anesthesia: a randomized controlled study. Drug Des Devel Ther..

[15] Mentz R.J., Hernandez A.F., Berdan L.G., Rorick T., O'Brien E.C., Ibarra J.C. (2016). Good clinical practice guidance and pragmatic clinical trials: balancing the best of both worlds. Circulation.

[16] Crellin D.J., Harrison D., Santamaria N., Babl F.E. (2015). Systematic review of the Face, legs, Activity, cry and consolability scale for assessing pain in infants and children: is it reliable, valid, and feasible for use?. Pain.

[17] Yao Y., Sun Y., Lin J., Chen W., Lin Y., Zheng X. (2020). Intranasal dexmedetomidine versus oral midazolam premedication to prevent emergence delirium in children undergoing strabismus surgery: a randomised controlled trial. Eur J Anaesthesiol.

[18] Tang S., Liu J., Ding Z., Shan T. (2024). The effect of dexmedetomidine on emergence delirium of postanesthesia events in pediatric department: a systematic review and meta-analysis of randomized controlled trials. Medicine (Baltimore).

[19] Gao J., Zheng Q., Liu M., Bao J. (2022). Functional Magnetic resonance imaging of brain function and emergence agitation of patients with dexmedetomidine-assisted general anesthesia under comfortable nursing intervention. Comput Intell Neurosci.

[20] Urits I., Peck J., Giacomazzi S., Patel R., Wolf J., Mathew D., Schwartz R. (2020). Emergence delirium in perioperative pediatric care: a review of current evidence and new directions. Adv Ther.

[21] Mason K.P. (2017). Paediatric emergence delirium: a comprehensive review and interpretation of the literature. Br J Anaesth.

[22] Larsen L.G., Wegger M., Lé Greves S., Erngaard L., Hansen T.G (2022). Emergence agitation in paediatric day case surgery: a randomised, single-blinded study comparing narcotrend and heart rate variability with standard monitoring. Eur J Anaesthesiol.

[23] Tang S., Liu J., Ding Z., Shan T. (2024). The effect of dexmedetomidine on emergence delirium of postanesthesia events in pediatric department: a systematic review and meta-analysis of randomized controlled trials. Medicine (Baltimore).

[24] Seo I.S., Seong C.R., Jung G., Park S.J., Kim S.Y., Kim M.M (2011). The effect of sub-tenon lidocaine injection on emergence agitation after general anaesthesia in paediatric strabismus surgery. Eur J Anaesthesiol.

[25] Tsuji Y., Koshika K., Ichinohe T. (2024). Effect of remimazolam and propofol anesthesia on autonomic nerve activities during Le Fort I osteotomy under general anesthesia: blinded randomized clinical trial. J Dent Anesth Pain Med.

