# Supplementary material for: Remimazolam for preventing sevoflurane-induced emergence delirium after pediatric laparoscopic inguinal hernia repair: a placebo-controlled randomized clinical trial
Source: J Pediatr (Rio J). 2025 Aug 22;101(5):101423. doi: 10.1016/j.jped.2025.101423 (PMC12495586; doi:10.1016/j.jped.2025.101423)
Supplement: Supplementary file 1 [file mmc1.docx]

**JPED-D-25-00098_ Supplementary Materials**

**Supplementary Table 1** Analysis of parental satisfaction and adverse events in the two groups [N, %].

|  | Control group  (N = 92) | Experimental group  (N = 92) | p-value |
| --- | --- | --- | --- |
| Parental satisfaction |  |  |  |
| Very satisfied with | 34 | 59 |  |
| Satisfied with | 43 | 19 |  |
| General | 6 | 9 |  |
| Not satisfied with | 5 | 5 |  |
| Very dissatisfied | 4 | 0 |  |
| The total satisfaction | 77 (83.69%) | 78 (84.78%) | 0.275 |
| Adverse events |  |  |  |
| Postoperative nausea or vomiting | 3 (3.26%) | 2 (2.17%) | 0.650 |
| Bradycardia | 0 | 0 |  |
| Hypotension | 0 | 0 |  |
| Laryngospasm | 0 | 0 |  |

**Supplementary Table 2** The specific scoring criteria and content of the PAED.

| Item | Description | Score |
| --- | --- | --- |
| Response to Environmental Stimuli | Insensitive | 0 |
|  | Slightly sensitive | 1 |
|  | Moderately sensitive | 2 |
|  | Very sensitive/Overreactive | 3 |
|  | Extremely sensitive/Uncontrollable reaction | 4 |
| Relationship with Parents or Caregivers | Maintains good relationship | 0 |
|  | Occasionally refuses contact | 1 |
|  | Frequently refuses contact | 2 |
|  | Almost always refuses contact | 3 |
|  | Completely uncommunicative/Refuses contact | 4 |
| Changes in Level of Consciousness | Alert and oriented | 0 |
|  | Slightly confused | 1 |
|  | Moderately confused | 2 |
|  | Significantly confused | 3 |
|  | Extremely confused/Disoriented | 4 |
| Ability to Focus Attention | Able to focus on tasks or people | 0 |
|  | Occasionally distracted | 1 |
|  | Frequently distracted | 2 |
|  | Almost always distracted | 3 |
|  | Completely unable to focus | 4 |
| Motor Activity | Calm | 0 |
|  | Slightly restless | 1 |
|  | Moderately restless | 2 |
|  | Significantly restless | 3 |
|  | Extremely restless/Constant activity | 4 |
